# Hdac1 and Hdac2 are essential for physiological maturation of a Cx3cr1 expressing subset of T-lymphocytes

**DOI:** 10.1186/s13104-021-05551-6

**Published:** 2021-04-13

**Authors:** Moumita Datta, Ori Staszewski

**Affiliations:** 1 Faculty of Medicine, Institute of Neuropathology, University of Freiburg, 79106 Freiburg, Germany; 2 Berta-Ottenstein-Programme for Clinician Scientists, Faculty of Medicine, University of Freiburg, 79106 Freiburg, Germany; 3 Present Address: Faculty of Medicine, Institute for Immunology, Ulm University, 89081 Ulm, Germany

**Keywords:** Cx3cr1, Hdac1, Hdac2, Epigenetics, T-Lymphocytes

## Abstract

**Objective:**

Histone acetylation is an important mechanism in the regulation of gene expression and plays a crucial role in both cellular development and cellular response to external or internal stimuli. One key aspect of this form of regulation is that acetylation marks can be added and removed from sites of regulation very quickly through the activity of histone acetyltransferases (HATs) and histone deacetylases (HDACs). The activity of both HATs and HDACs has been shown to be important for both physiological hematopoiesis as well as during development of hematological neoplasia, such as lymphomas. In the present study we analyzed the effect of knockout of the two HDACs, Hdac1 and Hdac2 in cells expressing the fractalkine receptor (Cx3cr1) on lymphocyte development.

**Results:**

We report data showing a maturation defect in mice harboring a Cx3cr1 dependent knockout of Hdac1 and 2. Furthermore, we report that these mice develop a T-cell neoplasia at about 4–5 months of age, suggesting that a Cx3cr1 expressing subpopulation of immature T-cells gives rise to T-cell lymphomas in the combined absence of Hdac1 and Hdac2.

**Supplementary Information:**

The online version contains supplementary material available at 10.1186/s13104-021-05551-6.

## Introduction

Histone deacetylases (HDACs) are part of the epigenetic regulation machinery and function as erasers of specific histone marks in tandem with histone acetyl transferases (HATs) to regulate gene expression [1]. HDACs are divided into 4 classes, with different substrate and target specificity [2]. The two ubiquitously expressed class I HDACs, Hdac1 and Hdac2 are highly homologous proteins, both of which are part of the repressive CoREST complex and play important roles in cell cycle regulation and cellular maturation [3, 4].

While Hdac1 and Hdac2 show high sequence homology and many redundant effects [3, 5], they do exhibit specific functions in various tissue types. Hdac1 activity is essential for embryonic development [6, 7], while Hdac2 activity is essential in pre- and postnatal tissue maturation, e.g., in brain development [7–9]. Inhibition as well as genetic ablation of both Hdac1 and Hdac2 lead to cell death by apoptosis in many, though not all, cell types [3, 10, 11]. In T-cells the activity of Hdac1 and Hdac2 is required for T-cell maturation, with ablation of either Hdac1 or Hdac2 in late stages of T-cell development leading to defects in the cluster of differentiation (CD) 4-positive to CD8-positive (CD4^+^/CD8^+^) ratio and to reduced anti-viral activity of CD8^+^ T-cells [12, 13]. Combined ablation in early stages of T-cell development leads to defects in the maturation of and genomic instability in double-positive T-cells [14].

The fractalkine receptor (Cx3cr1) is expressed on most myeloid cells and regarded as a myeloid cell marker. We could recently show that combined knockout of Hdac1 and Hdac2 genes in a Cx3cr1-dependent manner leads to maturation defects in microglia, the major brain resident myeloid cell type [15]. At the same time individual knockout of either Hdac1 or Hdac2 showed little effect on microglia. However, while Cx3cr1 expression is common in myeloid cells, a subset of both mature T- and B-cells express Cx3cr1 [16, 17]. In hematopoietic stem cells (HSCs), Cx3cr1-expression has not been reported and its expression is believed to be restricted to monocyte/macrophage and DC precursors (MDPs) [18, 19]. Here we report that Cx3cr1-dependent combined ablation of Hdac1 and Hdac2 leads to delayed maturation of T-cell precursors and to development of T-cell lymphomas.

## Main text

## Materials and methods

### Mice

All animal experiments were approved by the Ministry for Nature, Environment and Consumers’ Protection of Baden-Württemberg in accordance with EU directive 2010/63/EU and the German Animal Welfare Law under license G-12/103 and performed in accordance with the respective EU, federal and institutional regulations. Mice were housed in a specific pathogen free animal facility. They were group housed with up to five animals per cage and kept on a 12 h light/dark cycle. Food and water were accessible ad libitum. Cx3cr1-Cre Mice expressing the Cre-Recombinase under the Cx3cr1 promoter (Cx3cr1-Cre, official nomenclature B6J.B6N(Cg)-Cx3cr1tm1.1(cre)Jung/J) [18], Hdac1^loxP/loxP^ and Hdac2^loxP/loxP^ [3] mice were kept on a C57BL/6 background and crossed to generate mouse lines heterozygous for Cx3cr1-Cre and homozygous for both floxed Hdac1 and Hdac2. At 2 and 16 weeks of age mice were anesthetized and peripheral blood was obtained through a tail vein. Mice were then transcardially perfused with ice-cold phosphate-buffered saline (PBS). Tissue samples were removed for further processing for histology or flow cytometry. A total of 71 animals were analyzed, of these 10 (5 Cre+ animals and 5 Cre− littermates) were analyzed at 2 weeks and another 10 (5 Cre+ animals and 5 Cre− littermates) at 16 weeks of age with tissue samples taken for flow cytometry and histology as described below. All additional mice were observed until time of death or until they met humane end point definitions. Cre− animals not otherwise used in this study were removed from observation between 12 and 18 months of age.

### Fluorescence activated cell sorting (FACS)

Tissue was homogenized and homogenates as well as blood samples were treated with red blood cell (RBC) lysis buffer (BD). Cells were then washed with 0.5% fetal bovine serum (FBS) in PBS, counted and ~ 10^6^ cells were used per staining with the following antibodies: anti-CD11b (clone M1/70), anti-CD45 (clone 30-F11), anti-Thy1.2 (clone HIS51), anti-CD4 (clone GK1.5), anti-CD8 (clone 53-6.7), anti-CD24 (clone M1/69) and anti-T-cell-receptor beta (Tcrβ) (clone H57-597). Antibodies were purchased from eBiosciences. Cell counts were acquired on a FACS CANTO II system (Becton Dickinson) and data analyzed using FlowJo software.

### Histology & Immunohistochemistry

Histology and immunohistochemistry were performed as previously described [16]. After perfusion, organs were fixed in 4% paraformaldehyde in PBS. Samples were subsequently embedded in paraffin and stained with hematoxylin/eosin (H&E), MAC-3 (2.5 μg/ml, clone M3/84, BD Pharmingen), CD3 (3.5 μg/ml, clone CD3-12, Serotec), or B220 (2.5 μg/ml, clone RA3-6B2, BD Pharmingen) antibodies. For immunohistochemistry, 3 µm tissue sections were incubated overnight with the primary antibody at 4 °C and then with biotin-labeled goat anti-rat secondary antibody (2.5 μg/ml, Southern Biotech) for 45 min at room-temperature (RT). Streptavidin (Southern Biotech) incubation was performed for 45 min at RT and finally incubation with 3′-diaminobenzidine (DAB) brown chromogen (Dako) was performed.

### Data analysis

A priori sample size calculation was performed using GPower 3.1.9.2 [20]. For FACS and histological analysis a priori assumptions of effect size = 2, α = 0.05 and β = 0.75 were used, resulting in a group size of 5 per group. For survival analysis an effect size of 0.7 was assumed instead, resulting in a group size of 30 per group. Two-tailed t-tests were used to compare Cre+ and wildtype groups using GraphPad Prism 5 software (GraphPad Software Inc.). Experimenters were blinded as to the genotype until after data compilation for each data set, except for acquisition of survival data in which genotype was known to experimenters after the endpoint for each animal.

## Results

**Fig. 1:**
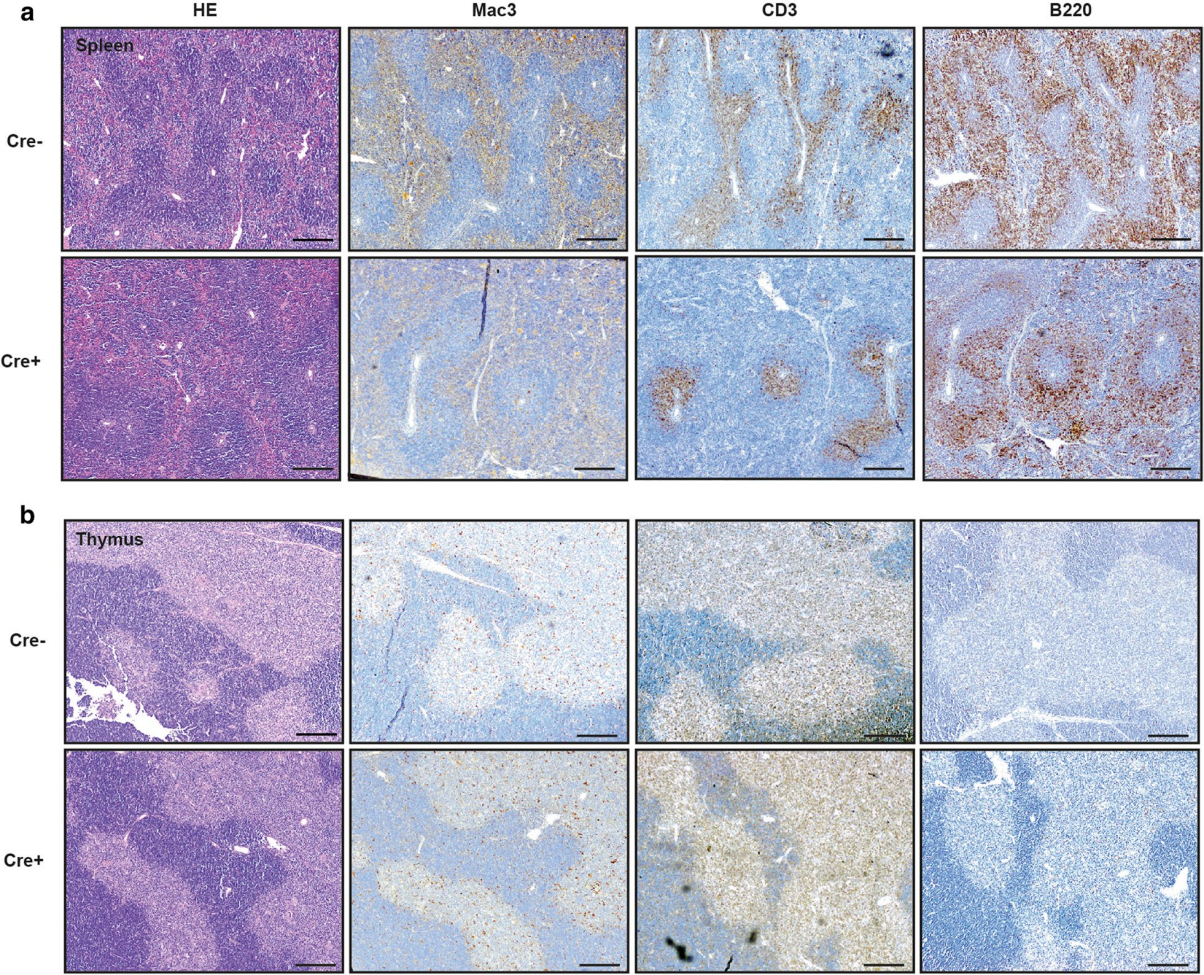
Histological analysis of the spleen (**a**) and thymus (**b**) of 2 week old mice with a combined Cx3cr1-dependent knockout of Hdac1 and 2 (Cre+) compared to Cre-negative littermates (Cre−). Histomorphology (HE) and cellular composition (macrophages (Mac3), T-lymphocytes (CD3) and B-lymphocytes (B220)) are depicted. Representative images of 5 Cre+ and 5 Cre− animals are shown. Scale bar = 500 µm

Mice with a combined Hdac1 and Hdac2 gene deletion in Cx3cr1 expressing cells were analyzed. These mice showed normal pre- and early postnatal development, except for previously reported alterations in brain microglia [15]. Lymphoid organs (thymus, lymph nodes and spleen) developed normally in these mice. At two weeks after birth no gross abnormalities in organ size or weight could be found in hematopoietic organs. Histological analysis of spleen (Fig. 1a) and thymus (Fig. 1b) also showed normal development. Other organs, such as heart, liver, lung, intestine and kidney likewise exhibited no gross or histological abnormalities. Cellular composition was also not altered; specifically macrophage and T- and B-cell numbers and distribution in the spleen and thymus did not differ between genotypes at 2 weeks of age (Fig. 1). Hdac expression in thymocytes was measured by Western blot and is provided in a Additional file 1: Supplementary Figure.

**Fig. 2:**
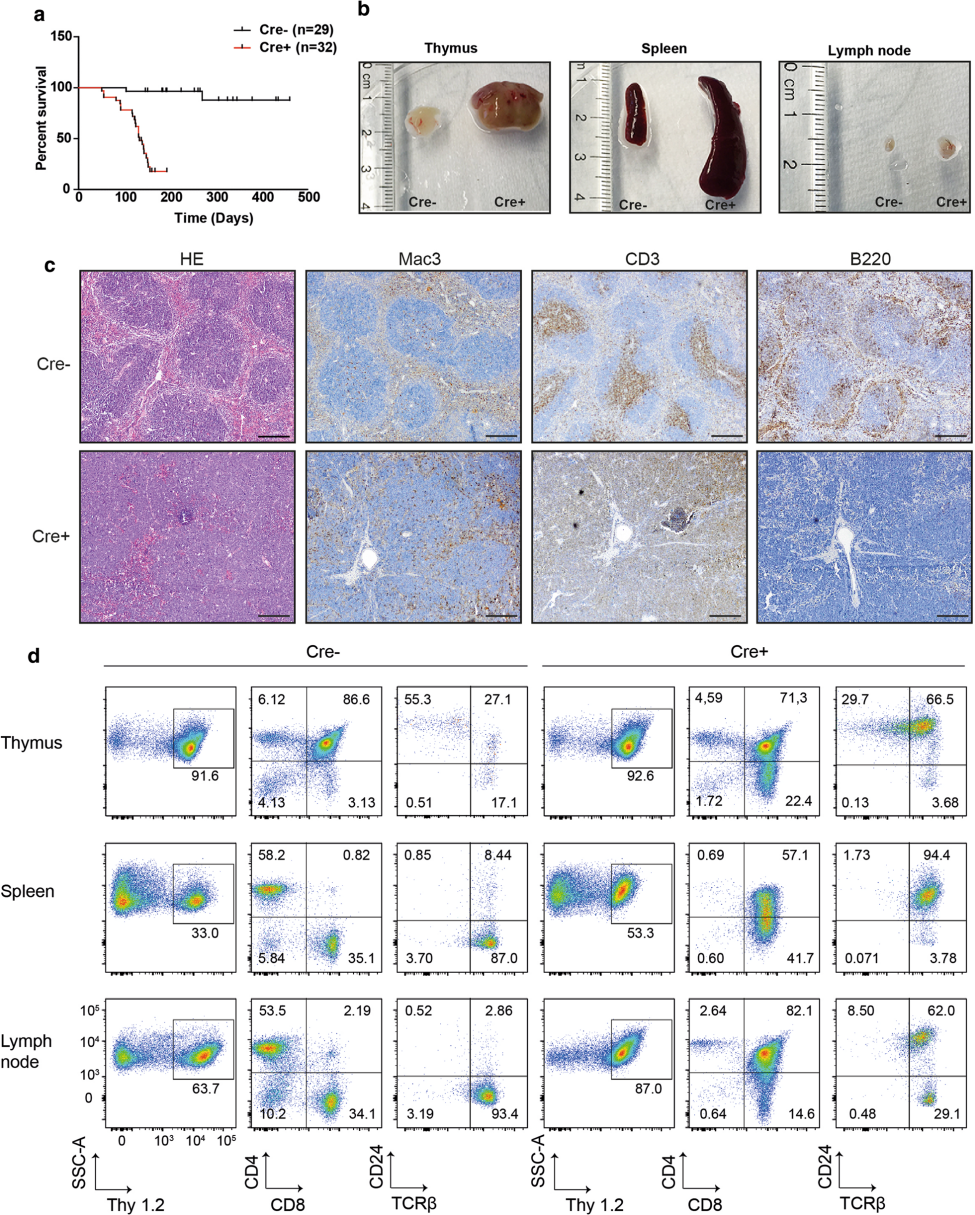
**a** Mice with combined knockout of Hdac1 and 2 (Cre+) show markedly reduced life span compared to wildtype littermates (Cre−). **b** At 16 weeks of age thymus, spleen and lymph nodes are strongly enlarged in knockout (Cre+) animals when compared to wildtype littermates (Cre−). **c** Histology and immunohistochemistry of 16 week old mice, comparing spleen samples of mice with combined Cx3cr1-dependent knockout of Hdac1 and 2 (Cre+) to their wildtype littermate (Cre−). Macrophages (Mac3), T-lymphocytes (CD3) and B-lymphocytes (B220) are depicted. Representative pictures of 5 Cre+ and 5 Cre−. Scale bar = 500 µm. **d** FACS analysis of cells isolated from thymus, spleen and lymph nodes from 16 week old Cre+ mice compared to their Cre− littermates. First column of each panel depicts gating for Thy1.2^+^ cells. The middle column shows CD4 vs CD8 fluorescence signal in the Thy1.2^+^ cells, while the right most column depicts CD24 vs Tcrβ fluorescence. Representative FACS plots for 5 replicates of each genotype are shown

At an age of 4–5 months all Hdac1/2 double knockout animals (Cre+) died, while wildtype littermates (Cre−) showed normal lifespan (Fig. 2a). Single knockout mice lacking either Hdac1 or Hdac2 exhibited no survival changes compared to wildtype animals. When Cre+ animals were euthanized at an age of 16 weeks, they harbored strongly enlarged thymus, spleen and lymph nodes compared to their Cre− littermates (Fig. 2b), while other organs appeared grossly normal (not shown).

Histological analysis of the enlarged organs revealed an almost complete destruction of the physiological structure of thymus and spleen with sheet-like expansion of lymphoid cells apparent throughout the organ (Fig. 2c). Immunohistochemical analysis of these altered tissue samples revealed an almost complete absence of B220^+^ B-lymphocytes in samples taken from 16 weeks old Cre+ mice, while the sheet like expansions consisted predominantly of CD3^+^ cells. Scattered macrophages were observed embedded within the tissue (Fig. 2c).

FACS of cells isolated from hematopoietic organs (spleen, thymus, lymph nodes) was performed to further analyze the lymphoid cell types altered within the enlarged tissue. As already apparent in histology, the percentage of Thy1 expressing T-cells was strongly increased in spleen (Fig. 2c, 2d), while it remained unchanged in thymus and lymph node samples. In all spleen and lymph nodes CD4/CD8 double-positive cell percentage and numbers increased markedly in 16 weeks old Cre+ mice compared to Cre− littermates. Additionally, CD8^+^ cells were found in increased percentages in thymus and spleen in Cre+ mice (Fig. 2d). Further analysis showed a marked increase in cells positive for both Tcrβ and CD24, marking the majority of Thy1 expressing cells in Cre+ mice as immature, proliferating T-cells (Fig. 2d).

**Fig. 3:**
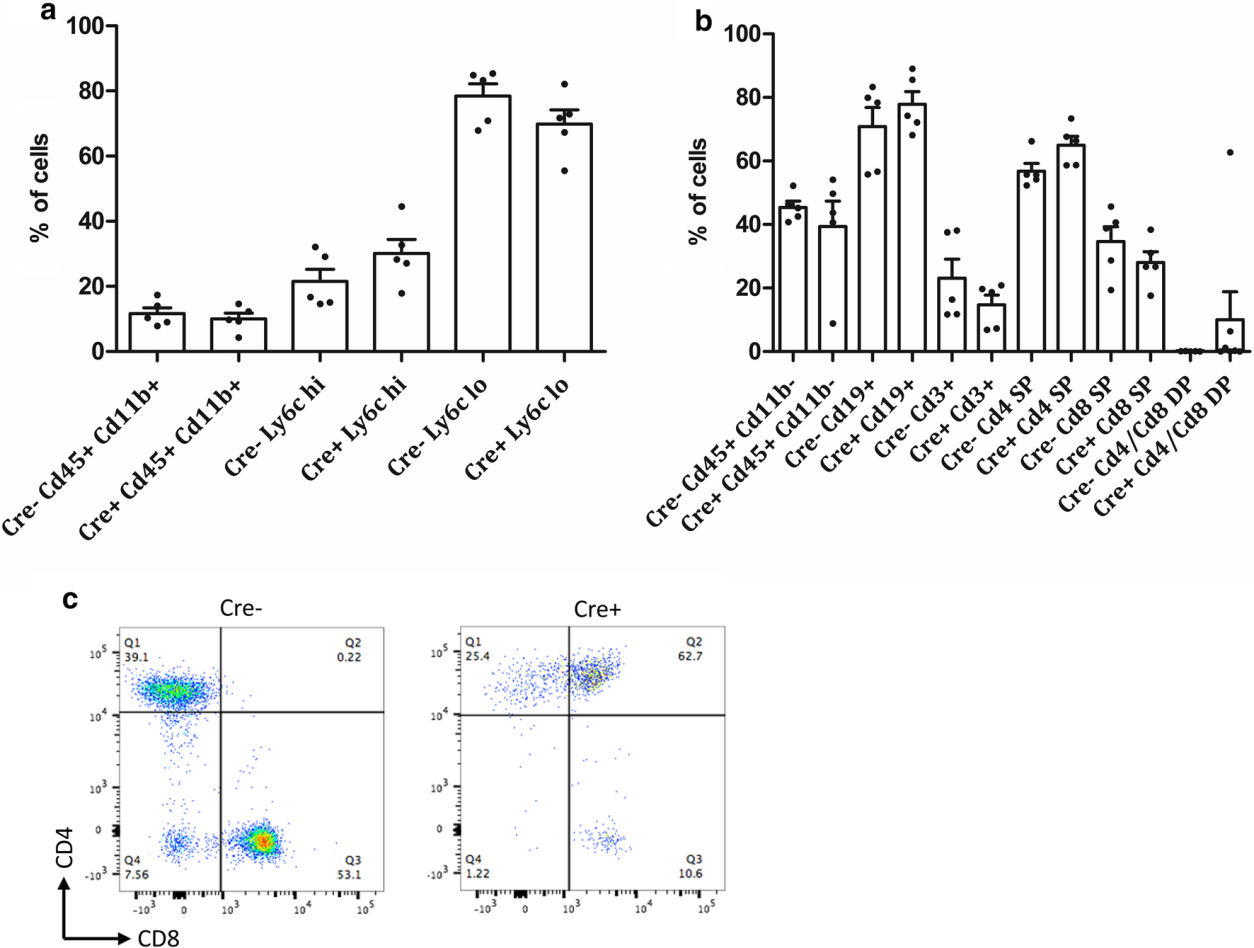
FACS quantification of blood samples taken from 16 week old mice with (Cre+) or without (Cre−) combined Hdac1 and 2 ablation. **a** Myeloid cells in peripheral blood: Cells were initially gated for CD45 and CD11b signal, percentages of CD45^+^ and CD11b^+^ cells on all cells are shown on the left. Of these CD45^+^ and CD11b^+^ cells, percentages of Ly6c-hi and Ly6c-low myeloid cells are shown. **b** Percentages of CD45^+^ and CD11b^−^ cells are shown on the left. Of these CD45^+^ and CD11b^−^ cells, percentages of CD19^+^ (B-) and CD3^+^ (T-)cells are shown next. To the right of these, the percentages of CD4 single-positive (SP), CD8 SP and CD4/CD8 double-positive (DP) cells compared to all CD3^+^ cells are depicted. No significant differences were found in either cell population, however some DP cells were seen in severely diseased mice. **c** FACS plot of a diseased Cre+ mouse, showing a large percentage of DP cells in blood, compared to a representative Cre− control

FACS analysis of blood samples of 16 week old mice was performed to elucidate changes in cell composition and in order to evaluate potential circulation of immature or neoplastic cells. Cellular composition appeared unaltered when comparing Cre+ mice to their wildtype littermates. Specifically neither B- nor T-cell numbers or percentages differed significantly between genotypes (Fig. 3a). When analyzing T-cell subsets, CD4^+^ and CD8^+^ T-cells, percentages also appeared unchanged (Fig. 3b). In wildtype mice no CD4/CD8 double-positive (DP) cells were found in peripheral blood. The same was true for some of the knockout mice analyzed, however while overall no significant differences were detectable, some mice that harbored very prominent neoplastic lesions upon histological analysis did show DP cells in blood (Fig. 3b, c).

We further analyzed liver, kidney, intestine and lung for possible spread of neoplastic cells. Neither of these organs showed macroscopic alterations in morphology, size or weight (not shown). Upon histological analysis no differences in morphology were found (not shown). Additionally, no immature or neoplastic lymphatic cell groups were evident in either of these non-lymphoid organs.

## Discussion

Expression of the chemokine receptor Cx3cr1 is commonly found in mature myeloid cells, such as monocytes, macrophages, dendritic cells and is utilized as a marker of myeloid cell populations. Furthermore Cx3cr1 expression in hematopoietic precursor cells is generally reported as a marker of myeloid lineage differentiation [19, 21]. While Cx3xr1 expression in mature lymphocytes has been reported in defined subsets of cells and increased under conditions causing lymphocyte activation [16, 22], no reports on its expression in lymphoid precursors have been made to date.

In the present study we report an effect of Hdac1 and Hdac2 ablation in Cx3cr1 expressing cells on T-cell maturation. Mice harboring a combined Hdac1 and Hdac2 deletion develop a T-cell neoplasia and exhibit a shortened life span compared to wildtype littermates. These mice further show strongly increased numbers of CD4/CD8 double-positive immature lymphocytes in secondary lymphoid organs (spleen, lymph nodes) combined with strongly increased expression of CD24, a marker of immature and proliferating lymphocytes, suggesting a delayed or impaired maturation of these cells in the thymus [23, 24].

The maturation delay as well as the development of a T-cell neoplasia, appear to be similar to the results obtained when Hdac1 and Hdac2 were deleted using Lck (lymphocyte protein tyrosine kinase)-Cre [14]. In Lck-Cre mediated Hdac1 and Hdac2 deletion, a marked increase in CD8^+^ cells in the thymus combined with a strong increase in double-positive (DP) cells in spleen and lymph nodes was found as well. Similarly a T-cell neoplasia in thymus and spleen with high lethality at about 4 months of age was also reported by the authors.

Cx3cr1-Cre is commonly used to analyze effects of gene deletion in myeloid cells with off-target effects on lymphocytes generally reported as affecting only a small percentage of mature T-cells [25–27]. The data presented here suggests that Cx3cr1-Cre mediated recombination may target a much larger subset of both immature and mature T-lymphocytes than previously reported. As such, studies utilizing this common Cre line for analysis of the myeloid cell compartment should take into account the potential of altering T-lymphocyte function alongside the myeloid cell compartment and take steps to control for such effects. Specifically any effects of experimental gene recombination on lymphocytic cell populations should be evaluated alongside the myeloid cell populations that are typically the focus of studies utilizing the Cx3cr1-Cre mouse model.

### Limitations

Taken together the results reported here for Cx3cr1-Cre mediated Hdac1 and Hdac2 deletion are very reminiscent of the Lck-Cre mediated deletion data and suggest a deleterious effect on T-cell maturation and potentially on genomic integrity in immature T-cells, however further analysis would be needed to determine the precise effect on genomic integrity and the mechanisms involved in any such effect. Additionally, we report a lack of B220-positive B-cells which warrants further investigation.

## Supplementary Information

**Additional file 1: Figure S1** (A): Western Blot analysis of Hdac1 and Hdac2 expression in thymic T-cells isolated from Cre+ and Cre- animals. Left panel shows representative blot, right panel depicts quantification relative to GAPDH expression. The lack of Hdac2 reduction is similar to findings from the Lck-Cre model [14]. (B) Uncropped versions of blot images, each blot contained from left to right two Cre- followed by three Cre+ samples. Rectangles indicated the cropping region for A.

## Data Availability

All data used in this study is provided in the manuscript, FACS and survival raw data is available upon request to the corresponding author.

## Abbreviations

CD: Cluster of differentiation
Cre−: Wildtype mice
Cre+: HDAC1/2 double knockout mice
DAB: 3′-Diaminobenzidine
DP: Double positive
FACS: Fluorescence activated cell sorting
HATs: Histone acetyltransferases
HDACs: Histone deacetylases
Lck: Lymphocyte protein tyrosine kinase
PBS: Phosphate buffered saline
RBC: Red blood cell
RT: Room temperature

## References

[1] Yang XJ, Seto E (2007). HATs and HDACs: from structure, function and regulation to novel strategies for therapy and prevention. Oncogene.

[2] Haberland M, Montgomery RL, Olson EN (2009). The many roles of histone deacetylases in development and physiology: implications for disease and therapy. Nat Rev Genet.

[3] Yamaguchi T, Cubizolles F, Zhang Y, Reichert N, Kohler H, Seiser C (2010). Histone deacetylases 1 and 2 act in concert to promote the G1-to-S progression. Genes Dev.

[4] Jamaladdin S, Kelly RD, O'Regan L, Dovey OM, Hodson GE, Millard CJ (2014). Histone deacetylase (HDAC) 1 and 2 are essential for accurate cell division and the pluripotency of embryonic stem cells. Proc Natl Acad Sci USA.

[5] Wilting RH, Yanover E, Heideman MR, Jacobs H, Horner J, van der Torre J (2010). Overlapping functions of Hdac1 and Hdac2 in cell cycle regulation and haematopoiesis. EMBO J.

[6] Lagger G, O'Carroll D, Rembold M, Khier H, Tischler J, Weitzer G (2002). Essential function of histone deacetylase 1 in proliferation control and CDK inhibitor repression. EMBO J.

[7] Dovey OM, Foster CT, Cowley SM (2010). Histone deacetylase 1 (HDAC1), but not HDAC2, controls embryonic stem cell differentiation. Proc Natl Acad Sci USA.

[8] Hagelkruys A, Lagger S, Krahmer J, Leopoldi A, Artaker M, Pusch O (2014). A single allele of Hdac2 but not Hdac1 is sufficient for normal mouse brain development in the absence of its paralog. Development.

[9] Montgomery RL, Davis CA, Potthoff MJ, Haberland M, Fielitz J, Qi X (2007). Histone deacetylases 1 and 2 redundantly regulate cardiac morphogenesis, growth, and contractility. Genes Dev.

[10] Zhou H, Cai Y, Liu D, Li M, Sha Y, Zhang W (2018). Pharmacological or transcriptional inhibition of both HDAC1 and 2 leads to cell cycle blockage and apoptosis via p21(Waf1/Cip1) and p19(INK4d) upregulation in hepatocellular carcinoma. Cell Prolif.

[11] Lin CL, Tsai ML, Lin CY, Hsu KW, Hsieh WS, Chi WM, et al. HDAC1 and HDAC2 double knockout triggers cell apoptosis in advanced thyroid cancer. Int J Mol Sci. 2019;20(2). 10.3390/ijms20020454 PMC6359659 30669676

[12] Boucheron N, Tschismarov R, Goeschl L, Moser MA, Lagger S, Sakaguchi S (2014). CD4(+) T cell lineage integrity is controlled by the histone deacetylases HDAC1 and HDAC2. Nat Immunol.

[13] Tschismarov R, Firner S, Gil-Cruz C, Goschl L, Boucheron N, Steiner G (2014). HDAC1 controls CD8+ T cell homeostasis and antiviral response. PLoS ONE.

[14] Dovey OM, Foster CT, Conte N, Edwards SA, Edwards JM, Singh R (2013). Histone deacetylase 1 and 2 are essential for normal T-cell development and genomic stability in mice. Blood.

[15] Datta M, Staszewski O, Raschi E, Frosch M, Hagemeyer N, Tay TL (2018). Histone deacetylases 1 and 2 regulate microglia function during development, homeostasis, and neurodegeneration in a context-dependent manner. Immunity.

[16] Goldmann T, Wieghofer P, Muller PF, Wolf Y, Varol D, Yona S (2013). A new type of microglia gene targeting shows TAK1 to be pivotal in CNS autoimmune inflammation. Nat Neurosci.

[17] Lee M, Lee Y, Song J, Lee J, Chang SY (2018). Tissue-specific role of CX3CR1 expressing immune cells and their relationships with human disease. Immune Netw.

[18] Yona S, Kim KW, Wolf Y, Mildner A, Varol D, Breker M (2013). Fate mapping reveals origins and dynamics of monocytes and tissue macrophages under homeostasis. Immunity.

[19] Auffray C, Fogg DK, Narni-Mancinelli E, Senechal B, Trouillet C, Saederup N (2009). CX3CR1+ CD115+ CD135+ common macrophage/DC precursors and the role of CX3CR1 in their response to inflammation. J Exp Med.

[20] Faul F, Erdfelder E, Lang AG, Buchner A (2007). G*Power 3: a flexible statistical power analysis program for the social, behavioral, and biomedical sciences. Behav Res Methods.

[21] Lyszkiewicz M, Witzlau K, Pommerencke J, Krueger A (2011). Chemokine receptor CX3CR1 promotes dendritic cell development under steady-state conditions. Eur J Immunol.

[22] Bottcher JP, Beyer M, Meissner F, Abdullah Z, Sander J, Hochst B (2015). Functional classification of memory CD8(+) T cells by CX3CR1 expression. Nat Commun.

[23] Xu X, Zhang S, Li P, Lu J, Xuan Q, Ge Q (2013). Maturation and emigration of single-positive thymocytes. Clin Dev Immunol.

[24] Li O, Zheng P, Liu Y (2004). CD24 expression on T cells is required for optimal T cell proliferation in lymphopenic host. J Exp Med.

[25] Abram CL, Roberge GL, Hu Y, Lowell CA (2014). Comparative analysis of the efficiency and specificity of myeloid-Cre deleting strains using ROSA-EYFP reporter mice. J Immunol Methods.

[26] Shi J, Hua L, Harmer D, Li P, Ren G (2018). Cre driver mice targeting macrophages. Methods Mol Biol.

[27] Jung S, Aliberti J, Graemmel P, Sunshine MJ, Kreutzberg GW, Sher A (2000). Analysis of fractalkine receptor CX(3)CR1 function by targeted deletion and green fluorescent protein reporter gene insertion. Mol Cell Biol.

