# Supplementary figures and images for: Hdac1 and Hdac2 are essential for physiological maturation of a Cx3cr1 expressing subset of T-lymphocytes
Source: BMC Res Notes. 2021 Apr 13;14:135. doi: 10.1186/s13104-021-05551-6 (PMC8045300; doi:10.1186/s13104-021-05551-6)

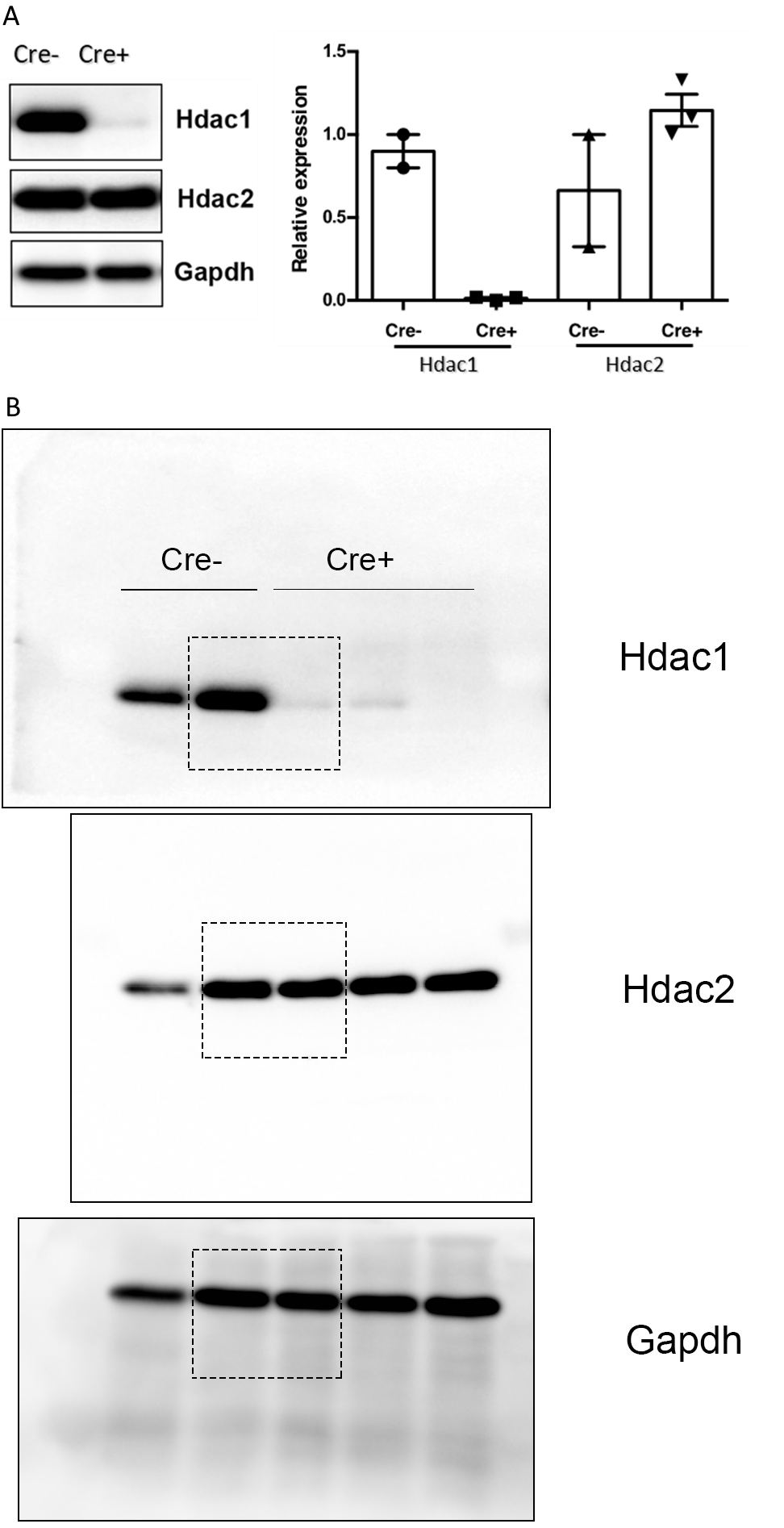

Supplement: Supplementary file 1 — Additional file 1: Figure S1 (A): Western Blot analysis of Hdac1 and Hdac2 expression in thymic T-cells isolated from Cre+ and Cre- animals. Left panel shows representative blot, right panel depicts quantification relative to GAPDH expression. The lack of Hdac2 reduction is similar to findings from the Lck-Cre model [14]. (B) Uncropped versions of blot images, each blot contained from left to right two Cre- followed by three Cre+ samples. Rectangles indicated the cropping region for A. [file 13104_2021_5551_MOESM1_ESM.tiff]
